# Evaluation of amino acid profile in serum of patients with Covid-19 for providing a new treatment strategy

**DOI:** 10.5937/jomb0-37514

**Published:** 2022-10-15

**Authors:** Ali Ozturk, Nihayet Bayraktar, Mustafa Bayraktar, Bashar Ibrahim, Taylan Bozok, Ceylan Mehmet Resat

**Affiliations:** 1 Niğde Omer Halisdemir University, Faculty of Medicine, Department of Medical Microbiology, Niğde, Turkey; 2 Harran University, Faculty of Medicine, Department of Medical Biochemistry, Şanlıurfa, Turkey; 3 Yıdırım Beyazıt University, Faculty of Medicine, Department of Internal Medical, Ankara, Turkey; 4 Suleyman Demirel Universit, Faculty of Pharmacy Department of Pharmaceutical Microbiology, Isparta, Turkey; 5 Mersin University, Faculty of Medicine, Department of Medical Microbiology, Mersin, Turkey; 6 Harran University, Faculty of Medicine, Department of Infectious Diseases, Şanlıurfa, Turkey

**Keywords:** amino acid, Covid-19, Sars-CoV-2, treatment strategy, aminokiselina, Covid-19, Sars-CoV-2, strategija lečenja

## Abstract

**Background:**

Amino acids have an important role in metabolism and may affect COVID-19-related outcomes. In our study, the amino acid serum level of hospitalized COVID19 patients was evaluated to determine a new treatment strategy.

**Methods:**

The amino acid profile covering 43 amino acids in 68 subjects, comprising 30 (14 men and 16 women) controls and 38 (16 men and 22 women) COVID-19 patients, were examined. The amino acid profiles of the participants were screened by LC-MS/MS.

**Results:**

Compared with the control group, serum levels of 27 amino acids increased in the patient group. Alpha-aminopimelic acid, sarcosine, and hydroxyproline amino acids were considerably higher in the control group than in the patient group (p<0.0001). There was no notable difference among control group and the case group for 13 amino acids (p≥0.05). A significant positive correlation was seen among the control and the patient groups in the mean amino acid values (r=0.937; p<0.0001).

**Conclusions:**

These results postulated a clear picture on the serum levels of amino acid in the COVID-19 patients. Serum amino acids measured in hospitalized COVID-19 patients can explain the patient's metabolic status during the disease.

## Abstract (sr)

**Uvod:**

Aminokiseline imaju važnu ulogu u metabolizmu i mogu uticati na ishode povezane sa COVID-19. U našoj studiji, nivo aminokiselina u serumu hospitalizovanih pacijenata sa COVID-19 procenjen je za određivanje nove strategije lečenja.

**Metode:**

Ispitivan je profil aminokiselina koji obuhvata 43 aminokiseline kod 68 ispitanika, od kojih su 30 (14 muškaraca i 16 žena) kontrolnih grupa i 38 (16 muškaraca i 22 žene) pacijenata obolelih od COVID-19. Profili aminokiselina učesnika su pregledani pomoću LC-MS/MS.

**Rezultati:**

U poređenju sa kontrolnom grupom, nivoi 27 aminokiselina u serumu su povećani u grupi pacijenata. Alfaaminopimelna kiselina, sarkozin i hidroksiprolin aminokiseline su bile znatno veće u kontrolnoj grupi nego u grupi pacijenata (p<0,0001). Nije bilo značajne razlike između kontrolne grupe i grupe slučaja za 13 aminokiselina (p≥0,05). Uočena je značajna pozitivna korelacija između kontrolne i grupe pacijenata u srednjim vrednostima aminokiselina (r=0,937; p<0,0001).

**Zaključak:**

Ovi rezultati su postavili jasnu sliku o serumskim nivoima aminokiselina kod pacijenata sa COVID-19. Aminokiseline u serumu merene kod hospitalizovanih pacijenata sa COVID-19 mogu da objasne metabolički status pacijenta tokom bolesti.

## Introduction

Severe acute respiratory syndrome coronavirus 2 (SARS-CoV-2), which emerged in China in the last months of the year 2019, has caused a worldwide pandemic in a short time as a highly pathogenic and contagious virus [1]. The coronavirus (COVID-19) pandemic due to severe acute respiratory syndrome coronavirus 2 (SARS-CoV-2) has affected more than 481 million people worldwide and killed more than 6 million people as of March 29, 2022 (WHO, 2022). The virus named SARS-CoV-2 by the World Health Organization (WHO) has been reported to be responsible for the 2019 coronavirus disease (COVID-19) [2].

The clinical manifestations of COVID-19 extend from asymptomatic to acute respiratory distress syndrome and multi-organ failure. In COVID-19 cases, fever, dry cough, myalgia, sore throat, fatigue, headache, dyspnea, diarrhea, and bilateral groundglass opacities are seen in chest CT scans [3]. COVID-19 is classified according to its severity as moderate, severe and critical. The moderate disease is characterized by dry cough, nasal congestion, mild fever, sore throat, myalgia, headache and malaise [4].

The majority (81%) of the COVID-19 cases are mildly severe. Patients with moderate symptoms may recover after 1–2 weeks. Depending on the situation of people with moderate disease, it can also turn into serious or critical cases. Severely ill individuals have severe pneumonia. Clinical signs include dyspnea, tachypnea (respiratory rate >30/min), oxygen saturation (SpO_2_) 93%, respiratory distress, partial pressure of oxygen (PaO_2_) / fraction of inspired oxygen (FiO_2_) <300, and/or lung infiltrates greater than 50%. In critical cases, septic shock, respiratory failure and other organ failure require mechanical ventilation and treatment in the intensive care unit (ICU) [4]. In SARS-CoV-2, a specific adaptive immune response is required for the host to eliminate the virus during incubation and non-severe stages of the disease and thus prevent progression to acute stages. Nevertheless, the deformation of the immune response can promote the disease to progress to advanced levels and even results in death [5].

The immun system is activated by the pathogens. The activation of immun system causes a significant increase in the immune system's demand for energy-producing substrates (e.g., amino acids, glucose and fatty acids). Activation of the immune response induces the construction of lipid-derived mediators such as leukotrienes, prostaglandins and many different types of proteins, including immunoglobulins, cytokines, cytokine receptors, chemokines, acute phase proteins and adhesion molecules [6] [7].

Amino acids are an essential part of the most important groups of metabolites and act as precursors for various majör cellular components, such as proteins and nucleobases. Therefore, they can be excellent markers of diseases due to their involvement in synthesizing proteins and metabolic regulators [8]. Their chemical properties and compositions not only determine the structure and function of proteins but also regulate the metabolic pathways related with the illness. Since changes in amino acid levels play a role in forming many diseases, replacing their deficiencies is beneficial in treating disease conditions. Metabolomic studies in the last 20 years have shown important changes in serum amino acid levels in various diseases, including diabetes, cancer, chronic kidney disease and Alzheimer's disease [9] [10]. The potential to use plasma amino acids as a metabolic subgroup to define disease risk has been demonstrated [11]. In addition, there is evidence that low amino acid levels may be a marker of protein malnutrition. Branched-chain amino acids (BCAAs) have significant effects on protein synthesis, cytokine activation, Ab production, glucose metabolism, and neurotransmission through cellular mammalian target of rapamycin (mTOR) signaling [12]. Low level of arginine bioavailability plays a role in the development of endothelial dysfunction and T cell dysregulation. This condition also leads to the development of the pathophysiology of multiple diseases [13].

However, about on the possible role of arginine bioavailability in children's COVID-19 and multisystem inflammatory syndrome (MIS-C) evaluating studies are not enough. Accordingly, there is not enough information in the literature on how the serum profile of amino acids, which are the precursors of these and similar biomolecules, which play an essential role in the immune response, is affected SARS-CoV-2 disease. Therefore, the amino acid levels in the serum of patients with COVID-19 were evaluated in this study. These biomarkers can help understand the pathophysiological process of COVID-19 disease and may have a special place for disease risk assessment.

## Materials and methods

### Study design

This study was conducted with hospitalized COVID-19 patients admitted to Niğde Ömer Halisdemir University Training and Research Hospital between March 11 and June 30, 2020, and diagnosed with the SARS-CoV-2 Reverse-Transcriptase Polymerase Chain Reaction (RT-PCR) test. A total of 38 (16 men and 22 women) adult patients diagnosed with COVID-19 by reverse transcription polymerase chain reaction (RT-PCR) test and receiving inpatient treatment were selected. In addition, a total of 30 subjects (14 men and 16 women) with negative RT-PCR tests and no chronic disease were included in the study as a healthy control group. In addition, all demographic, clinical, and microbiological data were obtained from the clinical records of the patients.

### SARS-CoV-2 RT-PCR test

A viral nucleic acid isolation kit was used to isolate SARS-CoV-2 from oro-nasopharyngeal swab and tracheal aspirate (TA) samples. In accordance with the manufacturer's recommendations (Biospeedy-Turkey), a sample of 10 μL (final volume) was used. RT-PCR kit (Biospeedy-Turkey) targeting N and ORF1ab genes of SARS-Cov 2 was used. Amplification was performed on the Qiagen Rotor-Gene Q 5plex HRM instrument (Qiagen, Germany).

### Analysis of serum amino acids by LC MS/MS

In this study, 43 amino acid levels were measured in the serum samples of the patient and control groups. After overnight fasting, 5 mL blood samples were taken from the patients and added to the tubes. The tubes were centrifuged at 5000 rpm for 10 min. After centrifugation, the serum samples were then stored in an Eppendorf tube at -80 °C until analysis. The analysis of serum samples was performed using the JASEM amino acid kit in the Biochemistry Laboratory of Harran University, Research and Practice Hospital of Faculty of Medicine, Turkey. The amino acid profile was measured using LC-MS/MS instrument (Shimadzu 8045, Japan). The experiment was performed by adding 350 μL and 25 μL internal standard on the cell pellet and mixed for 5 sec. The supernatant of 150 to 200 μL after centrifuging at 1000 g for 10 min was taken into insertable vial bottles and tested thrice using 8045 LC-MS/MS.

The sample was placed onto the tray in the High Performance Liquid Chromatography (HPLC) section of LC-MS/MS (Shimadzu 8045, Japan) device, and 43 different amino acid (Alanine, arginine, asparagine, aspartic acid, citrulline, glutamine, glutamic acid, glycine, histidine, leucine, isoleucine, alloisoleucine, lysine, methionine, ornithine, phenylalanine, proline, serine, threonine, tryptophan, tyrosine, valine, alpha-aminoadipic acid, alpha-aminopimelic acid, anserine, argininosuccinic acid, alpha-aminobutyric acid, beta-aminoisobutyric acid, gamm-aminobutyric acid, beta-alanine, sarcosine, cystathionine, thiaproline, 1-methylhistidine, 3methylhistidine, hydroxylysine, hydroxyproline, cystine, serotonin, histamine, etanolamine, 5-OH-Trp, taurine) were analyzed. An optical density (OD) for each amino acid was determined by reading the plates at 450 nm.

### Statistical analysis

The data analysis was carried out using SPSS V23 (IBM SPSS Statistics for Windows, Version 23.0. Armonk, NY: IBM Corp), and it was worked with a 95% confidence level. Shapiro Wilk test was performed for the normality test. Independent variable t-test was applied to those with normal distribution, while Mann U Whitney test was used for those who did not demonstrate normal distribution. Statistics were given for mean and standard deviation (mean ± SD) for numerical (quantitative) variables. Correlation analysis of variables was performed by Spearman analysis. Results were expressed as mean ± standard deviation and considered statistically significant at *p *<0.05 and *p*<0.001.

## Results

### General characteristics of groups

This study included 68 volunteers divided into two groups. The first group consisted of 38 volunteer patients with COVID-19, and the second group consisted of 30 healthy individuals as the control group.

The clinical features of all the participants are summarized in Table 1. The median age in the case group (n=38) included in the study was 61 (min-max: 30–95) years, while the median age in the control group (n=30) was 58.5 (min-max: 37–82) years. According to gender distribution, 57.9% of the case group was female (n=22) and 42.1% (n=16) was male, while 53.3% (n=16) of the control group was female and 46.7% (n=14) was male. 34.2% (n=13) of the patients were followed up in the intensive care unit, and 65.8% (n=25) in the wards.

**Table 1:** Demographic and clinical information of the patients with COVID-19 (n=38) HT, hypertension; COPD, chronic obstructive pulmonary disease; CKD, chronic kidney disease; DM, Diabetes mellitus

Variable	No.	%
Gender (male/ female)	16/22	42.1/57.9
Age (median (min-max))	61 (30 –95)	-
Days from disease onset to admission (mean±SD)	10.52±2.33	-
Symptoms of COVID-19		
Fever	25	65.8
Myalgia	15	39.5
Sore throat	16	42.1
Diarrhea	4	10.5
Dyspnea	9	23.7
Nausea	7	18.4
Headache	8	21
Admission to ICU	13	34.2
Admission to ward	25	65.8
Mechanical ventilation	25	65.8
High-flow nasal cannula	13	34.2
Comorbidities		
HT	5	13.1
COPD	6	15.8
Asthma	2	5.3
CKD	3	7.9
DM	3	7.9
Heart failure	2	5.3
Atrial fibrillation	1	2.6
Outcome (recovered)	27/11	71.0/29.0

### Amino acid profiles

Alanine, arginine, asparagine, aspartic acid, citrulline, glutamine, glutamic acid, glycine, histidine, leucine, isoleucine, alloisoleucine, lysine, methionine, ornithine, phenylalanine, proline, valine, alpha-aminoadipic acid, anserine, beta-aminoisobutyric acid, beta-alanine, 1-methylhistidine, 3-methylhistidine, serotonin, ethanolamine, and taurine amino acids were found to be significantly higher in the patient group than in the control group (*p*<0.05). Alpha-aminopimelic acid, sarcosine, and hydroxyproline amino acids were significantly higher in the control group than in the patient group (*p*<0.0001). There was no significant difference between the case and control groups for serine, threonine, tryptophan, tyrosine, argininosuccinic acid, alpha-aminobutyric acid, gamma-aminobutyric acid, cystathionine, thiaproline, hydroxylysine, cystine, histamine, and 5-OH-Trp (*p *0.05). When the amino acid levels of the group requiring intensive care and the group hospitalized in the ward were compared according to the type of hospitalization of the patients, glutamine (*p*=0.021), glutamic acid (*p*=0.004), histidine (*p*=0.044), serine (*p*=0.006), tryptophan (*p*=0.004), anserine (*p*=0.042) and taurine (*p*=0.049) levels were significantly different. Except for taurine, all of these amino acids were higher in intensive care patients, followed by the hospital ward patients.

A highly significant correlation was found between the control group and the samples in the mean amino acid values (r=0.937; *p*<0.0001) (Table 2).

**Table 2:** Amino acid mean values between the patient and control groups * Spearman correlation analysis was performed because the values showed non-parametric distribution<br> **Correlation is significant at the 0.01 level (2-tailed)

Correlations*				
			Controls	Samples
Spearman’s<br> rho	Controls	Correlation<br> Coefficient	1.000	0.937**
		Sig. (2-tailed)	NA	0.000
		N	43	43
	Samples	Correlation<br> Coefficient	0.937**	1.000
		Sig. (2-tailed)	0.000	NA
		N	43	43

## Discussion

Amino acids are the basis of proteins in our body. Amino acids are classified as essential, semi-essential, and non-essential amino acids. Our body needs twenty different amino acids. However, only 9 of them (isoleucine, leucine, lysine, methionine, phenylalanine, threonine, tryptophan, valine, and histidine) are necessary, and are taken from the outside as they are not synthesized in the human body. Depending on the pathological and physiological conditions of the individual, in addition to these 9 amino acids, arginine may be required to maintain body protein homeostasis and is called a semi-essential amino acid [14] [15] [16].

BCAAs, including leucine, valine and isoleucine are essential amino acids that have been studied in some disorders, particularly sepsis, liver cirrhosis, kidney failure, trauma, burn injury and cancer. For example, administration of an essential amino acid nutrient solution enriched with leucine rapidly activates the mTOR signaling pathway and protein synthesis in human skeletal muscle [17] [18].

In this context, amino acids provided from exogenous and/or endogenous proteins are of key importance for cell survival, continuity, and proliferation [19]. Mammals, in particular, have evolved biochemical and metabolic pathways to control pathogen infection by increasing amino acid catabolism and aid the immune response. This mechanism also provides the advantage for the host to control inflammatory responses to infections [20] [21]. Recent studies showed the role of amino acids in the regulation of immune response, particularly the activation of macrophages, B lymphocytes, T lymphocytes and natural killer cells. It has been demonstrated to have a key role in gene expression, cellular redox status, lymphocyte proliferation and the production of antibodies, cytokines, and other cytotoxic substances. Excess amino acid supply in the diet can be detrimental to the immune system due to the negative impact of amino acid imbalance and antagonism on food intake and use [22]. Many metabolic pathways are affected by the increase/decrease of plasma amino acid levels, as well as affect immune system, morbidity, and mortality [23].

Amino acids are recognized as mediators of metabolic cross-talk between host and pathogen. The host modifies metabolism to support defensive responses against the pathogen, while the pathogen uses metabolic signs to sense its anatomical location and immune status of the host [24]. However, the dynamics and clinical significance of changes in amino acid levels in patients with COVID-19 have not been adequately explained. Therefore, the purpose of this study, the effect of COVID-19 disease on serum amino acid levels was investigated. Studies have shown that changes in BCAA metabolism are common in many pathological conditions, and more studies are needed to better explain their therapeutic effects [9] [10]. In addition, studies have shown that those with low levels of essential amino acids are associated with anemia, cardiovascular disease, and infectious diseases [25].

The results of the present study postulate that the amino acid level are higher in the patient group than in the control group. In contrast, alpha-aminopimelic acid, sarcosine, and hydroxyproline amino acids were found to be lower. Higher levels of amino acids indicate chronic metabolic disorders. It has been observed that valine, leucine, and isoleucine amino acids can prevent cachexia, mostly during trauma, sepsis, and cancer. But, high levels of these amino acids are associated with metabolic encephalopathy, often with respiratory suppression, epileptic seizures, and brain damage due to lack of oxygen. In this context, the metabolic disorders of these amino acids need extracorporeal treatment not only during the diagnosis but also during the follow-up [26].

In the study of Atilla et al. [27] 2021 on COVID-19 patients, they reported that BCAAs did not detect significant differences between patient and control groups. However, according to the correlation analysis, they concluded that the correlation coefficients for leucine, isoleucine, and valine were greater than 0.8 and decreased together from the control to the severe patient group [27]. In our study, the seum BCAA level of the patients was increased compared to the control group, and this increase was found to be statistically significant. In the case of patients with myalgia complaints, the serum BCAA levels showed a significant increase, which may be due to increased muscle catabolism. However, the methodology and purpose of the present study are not suitable for making a definite judgment on this issue. Thus, further research was conducted on whether protein catabolism increases in the muscles of COVID-19 patients. While alanine is used as a substrate for gluconeogenesis by the liver, both alanine and glutamine are converted to tricarboxylic acid intermediates by leukocytes [28] [29].

In a study conducted in patients with sepsis, it was found that the serum alanine level increased, and this was due to BCAA metabolism [30]. In our study, the increase in serum alanine and glutamine levels in the patient group compared to the control group (*p*<0.0001) was significant (Table 3). In studies, it is known that the immune system is activated in the acute and convalescent period in Crimean-Congo Hemorrhagic Fever patients [29]. Although there is an increase in catabolic reactions in COVID-19 affected patients, the increase in serum glutamine and alanine levels suggested that there may be an increase in the use of glutamine and alanine during infection. The increase in the levels of these amino acids due to COVID-19 may be a pathology indication that results due to increasing protein breakdown in the lung tissue. A significant associations between inflammatory cytokine IL-6 levels with amino acid metabolism was observed in COVID-19 patients. This is due to certain effect of increased IL-6 on amino acids metabolism [31] [32]. In another study that some metabolites like anthranilic acid as effect of other inflammatory cytokine IL-18 led to increase in amino acid in critically COVID-19 patients [33]. In a similar study, increased in inflammatory and regulatory cytokines was found in severe cases of COVID-19 patients [34]. Cytokines were not measured in this study but alteration in levels of cytokines might result in alteration in amino acid levels either by protein degration or amino acid synthesis [31] [32] [33].

**Table 3:** Comparison of amino acids concentration in the healthy control group and patient with COVID-19 IQR=Interquartile Range (Q1–Q3); SD=Standard Deviation; *Normally distributed data

Amino acid	Pateint group (n=38)		Control group (n=30)		Significant level
	Median/Mean<br> μg/mL	IQR/SD	Median/Mean<br> μg/mL	IQR/SD	
Alanine	634.54	445.56–747	262.06	232.26–293.91	<0.0001
Arginine	142.90	93.8–170.04	66.99	59.1–72.31	<0.0001
Asparagine	83.90	52.74–109.78	42.79	37.17–48.7	<0.0001
Aspartic Acid	38.82	27.86–56.28	7.28	6.5–8.68	<0.0001
Citrulline	34.28	19.85–43.51	20.73	15.4–23.22	<0.0001
Glutamine	1112.88	821.59–1547.98	114.90	96.6–124.69	<0.0001
Glutamic Acid	145.91	93.46–172.41	69.83	61.92–76.98	<0.0001
Glycine	326.70	259.19–410.85	141.26	127.34–162.74	<0.0001
Histidine*	78.12	±26.77	56.72	±11.26	<0.0001
Leucine	213.13	165.26–278.06	90.78	83.09–109.34	<0.0001
Isoleucine	93.63	72.68–116.96	65.69	55.69–70.58	<0.0001
Alloisoleucine	0.73	0.59–0.92	0.37	0.31–0.43	<0.0001
Lysine	265.14	157.06–356.3	132.96	119.58–142	<0.0001
Methionine	34.27	22.7–46.45	24.55	21.26–27.18	0.003
Ornithine	145.59	98.23–227.13	64.23	55.13–91.18	<0.0001
Phenylalanine	132.16	110.21–175.78	50.92	42.48–59.02	<0.0001
Proline	262.77	183.37–389.63	148.30	124.57–187.46	<0.0001
Serine*	135.70	±47.67	130.52	±31.19	0.604
Threonine	140.14	105.18–212.17	136.60	93.54–157.91	0.283
Tryptophan	63.05	49.6–74.72	61.36	49.67–69.23	0.436
Tyrosine	76.53	60.45–110.91	67.15	60.52–78.74	0.056
Valine	299.74	229–398.38	154.85	138.78–170	<0.0001
Alpha-aminoadipic Acid	1.85	0.94–2.8	0.82	0.71–0.88	<0.0001
Alpha-aminopimelic Acid	0.72	0.66–0.79	44.11	40.93–45.86	<0.0001
Anserine	1.60	0.96–2.67	0.96	0.88–1.12	<0.0001
Argininosuccinic Acid	0.17	0.08–0.25	0.11	0.09–0.13	0.104
Alpha-aminobutyric Acid	18.40	11.19–31	15.73	14.09–17.54	0.277
Beta-aminoisobutyric Acid	5.60	3.45–6.69	2.87	2.29–3.56	<0.0001
Gamma-aminobutyric Acid	5.41	3.47–6.61	4.61	4.12–5.37	0.256
Beta-alanine	4.79	3.44–7.02	2.80	2.4–3.53	<0.0001
Sarcosine	34.84	24.4–43	47.38	38.93–63.5	<0.0001
Cystathionine	0.10	0.03–0.53	0.10	0.07–0.12	0.670
Thiaproline	0.05	0.02–0.42	0.10	0.09–0.12	0.408
1-Methylhistidine	5.23	3.34–7.25	1.34	1.06–1.51	<0.0001
3-Methylhistidine	4.22	1.93–5.97	0.21	0.17–0.26	<0.0001
Hydroxylysine	0.13	0.07–0.35	0.13	0.11–0.15	0.781
Hydroxyproline	8.17	6.35–9.64	28.58	24.03–31.93	<0.0001
Cystine	40.84	23.58–57.26	31.49	26.84–38.21	0.056
Serotonin	0.50	0.18–1.25	0.03	0.02–0.03	<0.0001
Histamine	0.02	0.01–0.03	0.01	0–0.03	0.240
Etanolamine	12.29	7.6–17.04	4.92	4.1–6.18	<0.0001
5-OH-Trp	0.03	0.02–0.04	0.02	0.01–0.03	0.060
Taurine	203.29	98.87–304.05	78.93	73.96–89.3	<0.0001

It is important to note that some amino acids, such as glutamine and alanine, play an important role in apoptosis inhibition. The previous study investigated the differences in plasma amino acid levels (PAA) between the acute and convalescent stages in individuals with community-acquired pneumonia (CAP), and healthy controls showed that plasma arginine and citrulline levels decreased.

However, in our current study, the levels of these amino acids were higher than in the control group. Our results show parallelism with the results of other studies [27]. Arginine is a substrate for nitric oxide (NO) production and is known to be involved in inflammation [35]. Citrulline and arginine play key roles in pathogenesis during bacterial infection. Arginine metabolism regulates host immunity. These results demonstrate that the metabolism of these amino acids in the urea cycle and NO production are significantly altered by altering host immunity during the acute phase of bacterial pneumonia [35] [36]. In this study, when the amino acid values of the groups hospitalized in the intensive care unit and the ward were compared, the amino acid levels showed a significant difference. A significant correlation was found between the control group and the samples in mean amino acid values (r=0.937; *p*<0.0001). The main limitation of our study is the relatively small sample size, mainly due to the limited availability of COVID-19 patients hospitalized during the pandemic. However, we believe this limitation will be overcome by implementing a longitudinal study design that allows detection of subject-specific effects while providing higher statistical power and minimizing potential interference with individual-level confounding variables such as age and gender. For better understanding the exact mechanisms underlying altered BCAA metabolism in COVID-19 and other diseases will occupy the mind of researchers for the foreseeable future.

## Conclusions

The present study shows that alterations not only one serum amino acids lenel but serveral amino acids may be of prognostic value in course of COVID-19 disease and they play an important role in its pathogenesis. It could also be suggested that the obtained data can be used as a biomarkers-treatment target in terms of the diagnosis of the disease, the course of the disease, and the effectiveness of the treatment. Specifically, the determination of amino acid profiles in patients with COVID-19 may be significant for developing different treatment strategies. Since there is not enough information about how the serum profile of amino acids is affected in SARS-CoV-2 disease, there is a need for new studies focusing on the serum amino acid profile. To better understand the disease, the present study results specifically recommend more studies to be planned to investigate the relationship between amino acids level and the immune system and further provide treatment strategies.

## Dodatak

### Author contributions

Conceptualization: AO and NB; methodology: NB, AO; validation: AO, NB; formal analysis: AO, BI, MRC and TB; investigation: NB; resources: AO,NB and MB; data curation, All author; manuscript writing: All author; visualization: AO and NB.

### Funding

The author(s) received no financial support for the research, authorship, and/or publication of this article.

### Ethical approval

This study was approved by the local ethics committee of Harran University, Faculty of Medicine, and the Ministry of Health, Turkey (Protocol no. HRU/21.16.20).

### Conflict of interest statement

All the authors declare that they have no conflict of interest in this work.

## References

[1] Cao Y, Cai K, Xiong L (2020). Coronavirus disease 2019: A new severe acute respiratory syndrome from Wuhan in China. Acta Virol.

[2] Ibrahim B, Önem E Coronavırus dısease 2019 (Covid-19): A lıterature revıew. Gevher Nesibe Journal IESDR.

[3] Harapan H, Itoh N, Yufika A, Winardi W, Keam S, Te H, et al (2020). Coronavirus disease 2019 (Covid-19): A literature review. J Infect Public Health.

[4] Ozturk A, Abdulmajed O, Ibrahim B (2020). What is the novel coronavirus disease 2019 (Covid-19) that paralyze the world?. Rev Med Microbiol.

[5] Shi Y, Wang Y, Shao C, Huang J, Gan J, Huang X, Bucci E, Piacentini M, Ippolito G, Melino G (2020). Covid-19 infection: The perspectives on immune responses. Cell Death Differ.

[6] Letelier P, Encina N, Morales P, Riffo A, Silva H, Riquelme I, Guzmán N (2021). Role of biochemical markers in the monitoring of COVID-19 patients. J Med Biochem.

[7] Abdulkhaleq L A, Assi M A, Abdullah R, Zamri-Saad M, Taufiq-Yap Y H, Hezmee M N M (2018). The crucial roles of inflammatory mediators in inflammation: A review. Vet World.

[8] Kim Y, Park S, Lee J, Jang J, Jung J, Koh J H, Choi C S, Wolfe R R, Kim I (2022). Essential amino acid-enriched diet alleviates dexamethasone-induced loss of muscle mass and function through stimulation of myofibrillar protein synthesis and improves glucose metabolism in mice. Metabolites.

[9] Kim I Y, Park S, Jang J, Wolfe R R (2020). Understanding muscle protein dynamics: Technical considerations for advancing sarcopenia research. Ann Geriatr Med Res.

[10] Bifari F, Nisoli E (2017). Branched-chain amino acids differently modulate catabolic and anabolic states in mammals: A pharmacological point of view. Br J Pharmacol.

[11] Borges A, Saavedra M, Simoes M (2015). Insights on antimicrobial resistance, biofilms and the use of phytochemicals as new antimicrobial agents. Curr Med Chem.

[12] Holeček M (2018). Branched-chain amino acids in health and disease: Metabolism, alterations in blood plasma, and as supplements. Nutr Metab (Lond).

[13] Rees C A, Rostad C A, Mantus G, Anderson E J, Chahroudi A, Jaggi P, Wrammert J, Ochoa J B, Ochoa A, Basu R K, Heilman S, Harris F, Lapp S A, Hussaini L, Vos M B, Brown L A (2021). Altered amino acid profile in patients with SARS-CoV-2 infection. Proc Natl Acad Sci U S A.

[14] Sakomura N K, Ekmay R D, Mei S J, Coon C N (2015). Lysine, methionine, phenylalanine, arginine, valine, isoleucine, leucine, and threonine maintenance requirements of broiler breeders. Poult Sci.

[15] Bröer S, Bröer A (2017). Amino acid homeostasis and signalling in mammalian cells and organisms. Biochemical Journal.

[16] Glenn J M, Madero E N, Bott N T (2019). Dietary protein and amino acid intake: Links to the maintenance of cognitive health. Nutrients.

[17] Church D D, Schwarz N A, Spillane M B, McKinley-Barnard S K, Andre T L, Ramirez A J, et al (2016). L-Leucine increases skeletal muscle Igf-1 but does not differentially increase Akt/mTORC1 Signaling and serum IGF-1 Compared to ursolic acid in response to resistance exercise in resistance-trained men. J Am Coll Nutr.

[18] Gwin J A, Church D D, Wolfe R R, Ferrando A A, Pasiakos S M (2020). Muscle protein synthesis and whole-body protein turnover responses to ingesting essential amino acids, intact protein, and protein-containing mixed meals with considerations for energy deficit. Nutrients.

[19] Park S, Church D D, Azhar G, Schutzler S E, Ferrando A A, Wolfe R R (2020). Anabolic response to essential amino acid plus whey protein composition is greater than whey protein alone in young healthy adults. J Int Soc Sports Nutr.

[20] Miyajima M (2020). Amino acids: Key sources for immunometabolites and immunotransmitters. Int Immunol.

[21] Butler M, van der Meer L T, van Leeuwen F N (2021). Amino acid depletion therapies: starving cancer cells to death. Trends Endocrinol Metab.

[22] Grohmann U, Mondanelli G, Belladonna M L, Orabona C, Pallotta M T, Iacono A, Puccetti P, Volpi C (2017). Amino-acid sensing and degrading pathways in immune regulation. Cytokine Growth Factor Rev.

[23] Iacone R, Scanzano C, Santarpia L, Cioffi I, Contaldo F, Pasanisi F (2020). Macronutrients in parenteral nutrition: Amino acids. Nutrients.

[24] Ren W, Rajendran R, Zhao Y, Tan B, Wu G, Bazer F W, Zhu G, Peng Y, Huang X, Deng J, Yin Y (2018). Amino acids as mediators of metabolic cross talk between host and pathogen. Front Immunol.

[25] Yamakado M (2018). 'Aminoindex technology' for lifestylerelated disease risk screening. Ningen Dock International.

[26] Demir Köse M, Canda E, Kağnıcı M, Altınok A Y, Uçar K S, Habif S, et al (2016). Branched-chain aminoacidopathies: Our experience in Ege University Faculty of Medicine. The Journal of Pediatric Research.

[27] Atila A, Alay H, Yaman M E, Akman T C, Cadirci E, Bayrak B, et al (2021). The serum amino acid profile in Covid-19. Amino Acids.

[28] Cruzat V, Rogero M M, Keane K N, Curi R, Newsholme P (2018). Glutamine: Metabolism and immune function, supplementation and clinical translation. Nutrients.

[29] Aydin H, Engin A, Kele S, Ertemur Z, Hekim N (2020). Glutamine depletion in patients with Crimean-Congo hemorrhagic fever. J Med Virol.

[30] Su L, Li H, Xie A, Liu D, Rao W, Lan L, Li X, Li F, Xiao K, Wang H, Yan P, Li X, Xie L (2015). Dynamic changes in amino acid concentration profiles in patients with sepsis. PLoS One.

[31] Thomas T, Stefanoni D, Reisz J A, Nemkov T, Bertolone L, Francis R O, Hudson K E, Zimring J C, Hansen K C, Hod E A, Spitalnik S L, D'Alessandro A (2020). Covid-19 infection alters kynurenine and fatty acid metabolism, correlating with IL-6 levels and renal status. JCI Insight.

[32] Masoodi M, Peschka M, Schmiedel S, Haddad M, Frye M, Maas C, Lohse A, Huber S, Kirchhof P, Nofer J R, Renné T (2022). Disturbed lipid and amino acid metabolisms in Covid-19 patients. J Mol Med (Berl).

[33] Danlos F X, Grajeda-Iglesias C, Durand S, Sauvat A, Roumier M, Cantin D, Colomba E, Rohmer J, Pommeret F, Baciarello G, Willekens C, Vasse M, Griscelli F (2021). Metabolomic analyses of Covid-19 patients unravel stage-dependent and prognostic biomarkers. Cell Death & Disease.

[34] Bayraktar N, Turan H, Bayraktar M, Ozturk A, Erdoğdu H (2022). Analysis of serum cytokine and protective vitamin D levels in severe cases of Covid-19. J Med Virol.

[35] Ikeda H (2021). Plasma amino acid levels in individuals with bacterial pneumonia and healthy controls. Clin Nutr ESPEN.

[36] Wijnands K A P, Castermans T M R, Hommen M P J, Meesters D M, Poeze M (2015). Arginine and citrulline and the immune response in sepsis. Nutrients.

